# Phenotypes and Genotypes of Old and Contemporary Porcine Strains Indicate a Temporal Change in the *S. aureus* Population Structure in Pigs

**DOI:** 10.1371/journal.pone.0101988

**Published:** 2014-07-07

**Authors:** Carmen Espinosa-Gongora, Arshnee Moodley, Urszula Lipinska, Els M. Broens, Katleen Hermans, Patrick Butaye, Luc A. Devriese, Freddy Haesebrouck, Luca Guardabassi

**Affiliations:** 1 Department of Veterinary Disease Biology, Faculty of Health and Medical Sciences, University of Copenhagen, Frederiksberg C, Denmark; 2 Department of Pathology, Bacteriology and Avian Diseases, Faculty of Veterinary Medicine, Ghent University, Merelbeke, Belgium; 3 Veterinary Microbiological Diagnostic Centre, Department of Infectious Diseases and Immunology, Faculty of Veterinary Medicine, Utrecht University, Utrecht, The Netherlands; 4 Unit of General Bacteriology, Centrum voor Onderzoek in Diergeneeskunde en Agrochemie - Centre d'Etude et de Recherches Vétérinaires et Agrochimiques (CODA-CERVA), Brussels, Belgium; Rockefeller University, United States of America

## Abstract

**Introduction:**

*Staphylococcus aureus* sequence type ST398 has recently gained attention due to the spread of methicillin-resistant strains among people exposed to livestock. The aim of this study was to explore temporal changes in the population structure of *S. aureus* in pigs over the last 40 years with particular reference to the occurrence of ST398.

**Methods:**

We analysed a unique collection of 91 porcine strains isolated in six countries between 1973 and 2009 using a biotyping scheme described in the 1970's in combination with *spa* typing and multi-locus sequence typing (MLST). The collection comprised 32 historical isolates from 1973–1974 (n = 19) and from 1991–2003 (n = 13), and 59 contemporary isolates from 2004–2009. The latter isolates represented the most common MLST types (ST1, ST9, ST97 and ST433) and *spa* types isolated from pigs in Europe.

**Results and Discussion:**

*S. aureus* sequence type ST398 was not found among old isolates from the 1970's or from 1991–2003, suggesting that this lineage was absent or present at low frequencies in pigs in the past. This hypothesis is supported by the observed association of ST398 with the ovine ecovar, which was not described in pigs by studies carried out in the 1970's. In addition, various phenotypic and genotypic differences were observed between old and contemporary isolates. Some biotypes commonly reported in pigs in the 1970's were either absent (human ecovar) or rare (biotype A) among contemporary isolates. Nine clonal lineages found among old porcine isolates are occasionally reported in pigs today (ST8, ST30, ST97, ST387, ST1092, ST2468) or have never been described in this animal host (ST12, ST133, ST1343). These results indicate that the population structure of porcine *S. aureus* has changed over the last 40 years and confirm the current theory that *S. aureus* ST398 does not originate from pigs.

## Introduction

*Staphylococcus aureus* is a ubiquitous bacterial species able to colonize and cause infections in a wide range of hosts. The host specificity of *S. aureus* was first investigated in the 1970's and 1980's by a series of studies based on biotyping [1]–[5]. These early studies described phenotypic differences between *S. aureus* isolated from humans and different animal species. Devriese [2] proposed a biotyping scheme based on four phenotypic tests to classify *S. aureus* lineages in biotypes. Some biotypes were regarded as host specific (ecovars), while others were regarded as non-host specific (NHS-biotypes). Over the years, this biotyping approach has been replaced by genotypic methods such as *spa* typing and multi-locus sequence typing (MLST), which have several advantages over phenotypic methods [6], [7]. To the best of our knowledge, possible associations between the biotypes described in the 1970's and the genotypes described nowadays in pigs have not been investigated prior to this study.

In recent years, *S. aureus* isolates belonging to sequence type (ST) 398 have gained interest as a zoonotic agent following the emergence of livestock-associated methicillin-resistant *Staphylococcus aureus*. The zoonotic potential of ST398 has been confirmed by several studies reporting infections in humans exposed to livestock [8]–[13]. This lineage is widespread in pigs [14]–[16] but has also been reported in other domestic animals such as cattle, broilers and horses [17]–[19]. Recently it has been hypothesized that ST398 originated from the human host and was transferred to livestock with subsequent gain of tetracycline and methicillin resistance [20].

The aim of the study was to identify possible changes in the population structure of *S. aureus* in pigs over the last 40 years with particular reference to the occurrence of ST398. For this purpose, a unique collection of 91 porcine strains collected from six countries between 1973 and 2009 was analysed using the old biotyping scheme described by Devriese [2] in combination with current genotypic methods.

## Materials and Methods

### Strains

We assembled a unique collection of 91 epidemiologically-unrelated porcine *S. aureus* isolates from six countries, including 32 historical isolates from 1973–1974 (n = 19) [2] and 1991–2003 (n = 13), and 59 contemporary isolates from 2004–2009 (Table 1). The historical collection covered all major biotypes described in pigs by Devriese and other authors investigating the diversity of *S. aureus* in the 1970's and 1980's [1]–[4]. To avoid overrepresentation of ST398 among contemporary isolates, we included 16 non-ST398 isolates belonging to all lineages described in pigs in Europe (ST1, ST9, ST97 and ST433) [15], [21] that were available within the networks of the four participating institutions (see acknowledgments). Within ST398, we selected 43 isolates based on *spa* type and country of origin to address as much as possible the diversity existing within this lineage. Some of these strains have been included in previous work [2], [15], [22]–[28].

**Table 1:** Year of isolation, genetic background and country of origin of the 91 *S. aureus* porcine isolates included in the study.

Years	*spa* types (n)	ST (CC)	Country*
1973–1974	t127 (1)	ST1 (CC1)	BE
	t008 (1)	ST8 (CC8)	
	t337 (1), t526 (3)	ST9, ST1092 (CC9)	
	t156 (1)	ST12 (CC12)	
	t012 (1), t318 (7)	ST30, ST2468 (CC30)	
	t1236 (1), t2112 (2)	ST387 (CC97)	
	t213 (1)	ST1343 (CC1343)	
1991–2003	t127 (1)	ST1 (CC1)	NL
	t337 (1), t899 (5), t1334 (1), t3446 (1),	ST9 (CC9)	DK, HK, NL
	t2112 (2)	ST97 (CC97)	NL
	t12057 (1)	ST133 (CC133)	NL
	t318 (1), t1130 (1), t1921 (1), t2840 (1), t3427 (3)	ST433, N.D. (CC30)	NL, DK
2004–2009	t127 (1)	ST1 (CC1)	NL
	t337 (2), t2498 (1)	ST9 (CC9)	DK, NL
	t318 (2), t1333 (3), t3427 (1)	ST433 (CC30)	NL, DK
	t011 (13), t034 (11), t108 (3), t567 (1), t571 (3), t899 (1), t1254 (1), t1255 (4), t1730 (1), t1793 (1), t2876 (2), t2922 (1), t4838 (1)	ST398 (CC398)	BE, CA, DK, IT, NL
	Total isolates 91		

N.D. ST unknown. The *spa* type was associated to CC30 on the basis of previous work [15], [29].

*Belgium (BE), Canada (CA), Denmark (DK), Hong Kong (HK), Italy (IT), The Netherlands (NL).

### Biotyping

All 91 isolates were biotyped following the method described by Devriese in 1984 [2]. The method includes four phenotypic tests: production of staphylokinase (K), β-haemolysin (β), coagulation of bovine plasma (BPC) and growth on crystal violet agar (CV) (Table 2). The combination of the test results generate four host-specific biotypes (ecovars) namely human, poultry, bovine and ovine, and five non-host-specific biotypes (NHS-biotypes) individually named after the test results: K− β+ CV:A (biotype A), K+ β− CV:A (biotype B), K+ β+ CV:A (biotype C), K− β− CV:C (biotype D) and K− β+ CV:C (biotype E). According to the original classification by Devriese, combinations of test results that did not correspond to any of the described biotypes were considered not allotted.

**Table 2:** *Staphylococcus aureus* biotypes as published by Devriese in 1984 [2].

Biotype*	Staphylokinase	β-haemolysin	Bovine plasma coagulation	Crystal violet growth type
**Host-specific ecovars**				
Human ecovar	+	−	−	C
Human ecovar β +	+	+	−	C
Poultry ecovar	−	−	−	A
Bovine ecovar	−	+	+	A
Ovine ecovar	−	+	+	C
**Non-host-specific (NHS) biotypes** **				
K− β+ CV:A	−	+	−	A
K+ β− CV:A	+	−	−	A
K+ β+ CV:A	+	+	−	A
K− β− CV:C	−	−	−	C
K− β+ CV:C	−	+	−	C

* Abbreviations derived from staphylokinase (K), β-haemolysin (β) and crystal violet growth type (CV).

** NHS-biotypes correspondence in this study: K− β+ CV:A (biotype A), K+ β− CV:A (biotype B), K+ β+ CV:A (biotype C), K− β− CV:C (biotype D) and K− β+ CV:C (biotype E).

### Genotyping

All isolates were *spa* typed as described previously [6]. MLST [7] was performed on 10 of the porcine isolates from the 1970's including one representative of each *spa* type found in this collection. ST398 isolates were confirmed by a CC398-specific PCR targeting *sau1-hsdS1* [18]. The remaining isolates were MLST-typed unless STs were available from previous studies [15], [23] or *spa*-ST associations were described in the scientific literature [15], [29]–[33]. MRSA isolates were identified by PCR detection of *mecA* [34].

## Results

Various remarkable differences were observed in the distribution of biotypes between porcine historical and contemporary isolates. The human ecovar and biotype B were only present in the historical collection (3 and 1 of 32 isolates, respectively), whereas biotype D was only present in the contemporary collection (1 of 59 isolates). Biotype E was the prevalent biotype in both collections and was associated to CC30 among historical isolates and to both CC30 and ST398 among contemporary isolates (Table 3). ST398 isolates belonged to either the ovine ecovar (23/43) or biotype E (20/43). None of the historical isolates from 1973–1974 belonged to the ovine ecovar. The only historical isolate displaying this biotype was a ST133 strain isolated in The Netherlands in 1991.

**Table 3:** Distribution of biotypes among *S. aureus* porcine isolates from the historical periods (1973–1974) and (1991–2003) and contemporary period (2004–2009) belonging to different sequence types (ST) or clonal complexes (CC).

Biotype	ST (*spa* types)	Time (n)
Human ecovar	ST8 (t008), ST12 (t156), ST2468/CC30 (t012)	1973–1974 (3)
Poultry ecovar	ST9 (t337), ST1092/CC9 (t526), ST1343/CC1343 (t213)	1973–1974 (5)
	ST9 (t337, t899)	2004–2009 (2)
Bovine ecovar	ST9 (t3446), ST97 (t2112)	1991–2003 (2)
	ST9 (t337, t899, t1334), ST9/CC9 (t2498)	2004–2009 (4)
Ovine ecovar	ST133 (t12057)	1991–2003 (1)
	ST398 (t011, t034, t571, t1254, t1255, t1793)	2004–2009 (23)
Biotype A	ST387/CC97 (t1236, t2112)	1973–1974 (3)
	ST1 (t127), ST97 (t2112)	1991–2003 (2)
	ST1 (t127)	2004–2009 (1)
Biotype B	ST1 (t127)	1973–1974 (1)
Biotype D	ST433/CC30 (t1333)	2004–2009 (1)
Biotype E	ST30 (t318)	1973–1974 (7)
	ST30 (t318), N.D./CC30 (t2840), ST433/CC30 (t1130, t1921, t3427)	1991–2003 (7)
	ST30/CC30 (t318), ST433/CC30 (t1333, t3427), ST398 (t034, t108, t567, t571, t899, t1255, t1730, t2876, t2922, t4838)	2004–2009 (7)
Not allotted	ST9 (t337)	1991–2003 (1)
	ST9 (t899)	2004–2009 (3)

N.D. ST unknown. The *spa* type was associated to CC30 on the basis of previous work [15], [29].

Thirty-two *spa* types associated with nine CCs were found among the 91 isolates tested. Twelve and four STs were detected among historical and contemporary isolates, respectively. The 19 isolates from the 1970's belonged to 10 *spa* types associated with nine STs and seven clonal complexes (Table 1). ST398 was not detected in the historical collection.

MRSA ST398 isolates were mainly represented by the ovine ecovar (21/32), whereas biotype E was prevalent among methicillin-susceptible isolates (9/11). MRSA ST9 isolates were not allotted (2/4) or belonged to the bovine (1/4) and poultry (1/4) ecovars.

## Discussion

The absence of *S. aureus* ST398 in the historical collection suggests that this lineage was absent or present at a low frequency during the 1970's and through the early 2000's. This hypothesis is further supported by the observation that the ovine ecovar, which was one of the two main biotypes associated with ST398 and the most prevalent among MRSA isolates, was neither found among the historical isolates from the 1970's nor reported in pigs by any of the studies conducted at that time in Belgium, Czechoslovakia, Russia and Greece [2], [5], [35], [36], even though in one of the studies the actual biotype of the isolates could not be defined retrospectively since the CV phenotype was not indicated in the original manuscript [36]. Our finding that ST398 is partly associated to a biotype that was not reported in pigs in the 1970's is in line with the hypothesis by Price et al [20] that this *S. aureus* lineage originated from humans.

Despite the small number of isolates representative of the 1970's, this unique collection contained all the major biotypes described in pigs in the past [1], [2], [5], [35] and all the most common clonal lineages reported in pigs today except ST398 [15], [37], [38]. However, marked phenotypic and genotypic differences were observed between historical and contemporary isolates, indicating that the *S. aureus* population structure in pigs has undergone major changes over the last 40 years. The most common biotypes among historical isolates were absent or rare among contemporary isolates and *vice versa* except for biotype E, which was prevalent in both groups of isolates. The two most common biotypes reported by Devriese in 1984 [2], biotype A (38/68 isolates) and the human ecovar (14/68 isolates), were represented in our historical collection (5/32 and 3/32 isolates, respectively) but were either rare (1/59 isolates belonging to biotype A) or absent (human ecovar) in our contemporary collection. A temporal reduction in the diversity of the porcine *S. aureus* population could be inferred on the basis of MLST since nine STs found among historical isolates could not be detected among contemporary isolates (Table 1), and are not currently associated with the pig host [15], [37], [38]. The temporal change in population structure was confirmed by *spa* typing since the *spa* types displayed by historical isolates are only occasionally reported in pigs today (t008, t012, t1236 and t526) [14], [15], [24], [38]–[40] or have only been reported in humans (t156 and t213) [41], [42]. Based on these data, it appears that ST398 could have displaced other lineages present in this animal reservoir in the past. This displacement is unlikely to be a direct consequence of antimicrobial use since tetracycline resistance, a typical feature of this lineage, was not uncommon among porcine isolates described in the 1970's [5] as confirmed by antimicrobial susceptibility testing of our collection (data not shown).

Interestingly, all biotypes associated with ST398 (ovine ecovar and biotype E) and the human ecovar share a common phenotype (CV type C). The assumption that ST398 has originated from humans reinforces an old hypothesis that this phenotypic trait might be conserved in *S. aureus* of human origin [43]–[45]. The human ecovar was exclusively found among isolates from the 1970's, suggesting a closer relationship between porcine and human *S. aureus* in those years. The human ecovar is characterized by production of staphylokinase (K+), which is encoded by *sak* as part of the human immune evasion cluster (IEC) in φSa3. This phage frequently inserts into the β-hemolysin gene (*hlb*) of human strains, thereby inactivating it [46]–[48]. Therefore, the presumed human origin of ST398 would have involved loss of this mobile genetic element to restore the phenotype K− β+ typical of the lineage [20], [49]. Other *S. aureus* lineages (e.g. CC30, CC9, CC97 and CC1) displayed two or more distinctive phenotypic traits (e.g. growth on CV agar) regardless of the time of isolation, indicating specific associations between genotypes and phenotypes.

The one and most important limitation of the study is the limited size of the strain collection, especially in relation to the small number of historical isolates from the 1970's. However, these ancient isolates retrieved from the Devriese's strain collection were representative of the diversity observed in pigs at that time. Moreover, our conclusion that 40 years ago ST398 either did not occur or was rare in pigs in Europe is not only supported by the absence of this lineage in the historical strain collection but also by the finding that most contemporary ST398 isolates display a biotype (i.e. the ovine ecovar) that has never been described in pigs in the past. The possibility that ST398 was present in pigs 30–40 years ago with a different biotype implies that this lineage underwent major changes to acquire the specific phenotype of the ovine ecovar (K− β+ BPC+ CV:C), including the phenotype on CV, which has been shown to be a stable phenotypic trait in this and other studies [44], [45]. Although distribution of *S. aureus* lineages is known to vary geographically, nowadays ST398 is widespread in the pig population in the two countries from which our historical isolates originated from (Belgium and The Netherlands) as well in other European countries where biotype distribution was studied in the 1970's [50]. Based on these considerations, we conclude that the outcome of the study could only be marginally influenced by the size and geographical origin of the strain collection.

## Conclusions

The *S. aureus* population in pigs today is different to that of 30–40 years ago. Some genetic lineages seem to have disappeared, probably displaced by the spread of ST398. The absence of ST398 or associated biotypes in the porcine isolates from the 1970's through early 2000's supports the current hypothesis that this lineage does not originate from pigs.
